# Health inequalities in cause-specific mortality in Costa Rica: a population-based cohort study

**DOI:** 10.11606/s1518-8787.2023057004331

**Published:** 2023-02-08

**Authors:** Romain Fantin, Cyrille Delpierre, Cristina Barboza-Solís

**Affiliations:** I Universidad de Costa Rica Centro Centroamericano de Población San José Costa Rica Universidad de Costa Rica. Centro Centroamericano de Población. San José, Costa Rica.; II Universidad de Costa Rica Facultad de Medicina Escuela de Salud Pública San José Costa Rica Universidad de Costa Rica. Facultad de Medicina. Escuela de Salud Pública. San José, Costa Rica; III Universidad de Costa Rica Facultad de Odontología San José Costa Rica Universidad de Costa Rica. Facultad de Odontología. San José, Costa Rica; IV Inserm-University Toulouse III Paul Sabatier Center for Epidemiology and Research in Population health Toulouse France Inserm-University Toulouse III Paul Sabatier. Center for Epidemiology and Research in Population health. Toulouse, France

**Keywords:** Cause of Death, Health Status Disparities, Socioeconomic Factors, Developing Countries, Ecological Studies

## Abstract

**OBJECTIVE:**

To analyze health inequalities in cause-specific mortality in Costa Rica from 2010 to 2018, observing the main causes for inequality in the country.

**METHODS:**

The National Electoral Rolls were used to follow-up all Costa Rican adults aged 20 years or older from 2010 to 2018 (n = 2,739,733) in an ecological study. A parametric survival model based on the Gompertz distribution was performed and the event death was classified according to the ICD-10.

**RESULTS:**

After adjustment for urbanicity, the poorest districts had a higher mortality than the wealthier districts for most causes of death except neoplasms, mental and behavioral disorders, and diseases of the nervous system. Urban districts showed significantly higher mortality than mixed and rural districts after adjustment for wealth for most causes except mental and behavioral disorders, diseases of the nervous system, and diseases of the respiratory system. Differences according to wealth were more frequent in women than men, whereas differences according to urbanicity were more frequent in men than in women.

**CONCLUSIONS:**

The study’s findings were consistent, but not fully similar, to the international literature.

## INTRODUCTION

Public health research^1^ has consistently found socioeconomic inequalities in mortality, showing a negative social gradient where those with lower socioeconomic positions (SEP) have higher mortality^2,3^. Largely studied in high-income countries^4^, health inequality in mortality persists across time^5^ and countries^6^. Additionally, in high-income countries, negative social gradients have been observed for many specific causes of death^7^. In Europe and the United States, for example, lower socio-economic groups were found to have higher cardiovascular disease mortality ^8,11^.

Nevertheless, health inequality differs with country and context^14^. In Northern European countries, mortality from cardiovascular disease is one of the main cause of death associated with socioeconomic inequalities^11^; in Southern European countries, mortality from cancer; and in Eastern European countries, mortality from accidents^17^. Other studies also showed that death rates and causes of death might be different between rural and urban areas. In the United States, women living in rural areas had far higher death rates for lung cancer and chronic obstructive pulmonary disease than women living in urban areas^18^.

Despite the extensive literature on socioeconomic inequalities in mortality, studies focused on data from low and middle-income countries are still incipient^19^, with no evidence for several Latin American countries. A study including various areas of Latin American countries found that education is a protective factor for mortality, showing significant differences between rural and urban characteristics^20^. In São Paulo (Brazil), differences according to income were found for homicide, ischemic heart disease, HIV/Aids, and respiratory diseases^21^. Finally, studies have also found differences in leading causes of death according to urbanicity. In Peru and in Mexico, for example, liver diseases were more prevalent in rural areas whereas strokes were more prevalent in urban areas^20^.

Health inequalities in mortality might partially differ across countries and cultural contexts because of the socioeconomic distribution of health behaviors and access to health care^17,22^ or the level of socioeconomic inequalities in the country^23^. This motivates researchers to analyze and report on different countries to better understand social determinants and reduce health inequalities in mortality. These studies should go beyond high-income countries^24^ since socioeconomic inequality in low and middle-income countries are a major political and public concern with individual and collective effects^23^.

Costa Rica is an interesting case. The middle-income Latin American country of 5 million inhabitants has significant socioeconomic inequalities^25^. However, life expectancy in the country is 80 years and Costa Ricans have similar health statistics to populations of high-income countries^13,26^. Moreover, the quality and completeness of the mortality data^27^ and the existence of periodic censuses for socioeconomic data allowed powerful ecological studies on the country. Finally, Rosero-Bixby and Dow explored why the USA had higher mortality rates than Costa Rica despite having a much higher GDP^13^: based on the 1984 Census, the difference was related to the USA’s “*much steeper socioeconomic gradients in health*” for heart diseases and diabetes. The USA also presented large inequality for nine studied causes of death, against only three causes in Costa Rica – cerebrovascular diseases, external injuries, and particularly chronic respiratory mortality)^13^.

Earlier studies suggested the inexistence of a negative social gradient in cancer^28,29^ and overall mortality^30,31^, diverging from the existing literature worldwide. However, increasing socioeconomic inequality can change the social gradient in mortality, as seen in a recent study on differences in life expectancy of people born in the province of Limon, one of the poorest of the country, and of people born in other provinces^32^.

Another study, which used an ecological design, also aimed to reveal socioeconomic inequality in mortality in Costa Rica^33^. The study analyzed wealth and urbanicity in the electoral district and included all adult Costa Rican citizens, reporting the importance of the adjustment for urbanicity. After the adjustment, the results showed that people living in poorer districts had higher mortality than those in wealthier districts. Moreover, overall mortality was lower in rural areas than in urban areas for men but not for women. Life expectancy was therefore similar in the poorest rural districts and in the richest urban districts for men, but two years higher in the richest urban districts than the poorest rural districts for women.

This complex interaction between sex, urbanicity, and wealth might explain why previous studies could not show a clear social gradient in mortality in Costa Rica. Nevertheless, the study provided no information on specific-cause mortality, which would allow better understanding of health inequalities in mortality in Costa Rica to effectively address health interventions.

This study aimed to analyze health inequalities in cause-specific mortality in Costa Rica from 2010 to 2018 to observe the main causes of inequality in the country.

## METHODS

### Sample

The sample was based on the National Electoral Rolls (NER) used for the presidential elections of 2010. NER included all adult Costa Rican citizens older than 18 years on January 1^st^, 2010 (N = 2,739,733). In our study, Costa Ricans over 20 years old were included. Foreign citizens are not included in the NER and were therefore excluded from this study. NER was merged with the National Death Index to find the date of death and with the registry of the National Institute of Statistics and Census to find the main cause of death. They were merged using the *cédula de identidad*, an indispensable unique identification number assigned to Costa Ricans at birth or upon naturalization.

The sample used in this article was first presented in previous studies. For further details, please see Fantin et al.^33^ and Barboza-Solís et al.^34^.

### Causes of Deaths

The National Institute of Statistics and Census (INEC in Spanish) registers all death certificates^27^, including the main cause of death as reported in the death certificate using the 10^th^ revision of the International Classification of Diseases (ICD-10). The sample previously described and the INEC registry were anonymously merged by sex, age at death, and characteristics of the electoral district. The ICD-10 chapters analyzed were those with more than 1,000 deaths during the studied period, such as: Certain infectious and parasitic diseases (A00–B99); neoplasms (C00–D48); endocrine, nutritional and metabolic diseases (E00–E90); mental and behavioral disorders (F00–F99); diseases of the nervous system (G00–G99; diseases of the circulatory system (I00–I99); diseases of the respiratory system (J00–J99); diseases of the digestive system (K00–K93); diseases of the genitourinary system (N00–N99); diseases of the musculoskeletal system and connective tissue (M00–M99); and external causes of morbidity and mortality (V01–Y98). Symptoms, signs and abnormal clinical and laboratory findings not elsewhere classified (R00–R99) and other, which included all chapters without at least 1,000 deaths, were presented to illustrate the consistency of the data.

### Socioeconomic Data

An ecological study was conducted at the level of the electoral district^22^. The smallest administrative division in Costa Rica, the district, was analyzed. Costa Rica has 5 million inhabitants and is administratively divided into 477 districts (data from 2015). The median population by district was 3,458 inhabitants ([quartiles 1=1,697, quartiles 3=7,085]). To describe the districts, we used the same approach of our previous study on social gradient in cancer mortality in Costa Rica^22^. Each district was described using the 2011 Census, which includes more than 4.3 million people (94% of the population in 2011). Districts were classified by urbanicity (urban, mixed, and rural) and wealth. A district is considered urban if more than 80% of its population lives in urban areas, rural if less than 20% of its population lives in urban areas, and mixed if otherwise^35^. Classification by wealth was based on the percentage of people with at least one Basic Unmet Need (BUN)^36^ according to the 2011 Census. INEC uses this indicator to measure poverty at the county or district level in four dimensions: access to decent shelter (building materials used and overcrowding), access to a healthy life (access to drinking water, sanitation, disposal of excreta), access to basic education (children’s school attendance), and access to other goods and services^34,36^. Districts were divided by BUN into quartiles of population according to the 2011 Census population in each district.

### Weighting

Although the INEC registered incorrectly the cédulas of 7,883 persons (4.9% of all death records), the total distribution of the cause of death and the district characteristics were known for this population. Since these incorrect cédulas were excluded, to account for possible bias, the merged death observations were weighted to reflect on average the sexes, ages, electoral district characteristics, and causes of death in the total sample. Using STATA, a calibrated function calculated the weights ranging from 1.03 to 1.10 for people who died in 2011-2018. This method was first described in a previous study^34^.

The sample included 2,739,733 people for 23,950,240 person-years of follow-up and 153,815 deaths. The weighted sample included 2,747,616 people and 161,698 deaths.

### Model

The outcome of interest was the time-to-event. For each specific cause of death, the event was death by the cause studied. People who died for a cause other than the one studied were considered as right-censored. For adjustment for age, the date of entry of each subject was their age on January 1st, 2010, or 20 years old. The date of the last follow-up was either the subject’s age at death or their age on December 31, 2018. A parametric survival model based on the Gompertz distribution was used. Cluster sampling considered electoral districts as clusters. The model was weighted.

The main analysis was a model adjusted for sex, urbanicity, and district wealth as a categorical variable. The Relative Index of Inequality (RII) with (RII_A_) and without adjustment for urbanicity (RII_NA_) were also presented. To calculate the RII, the categorical quartiles of wealth were used as a continuous variable in the model, ranging from 0 to 1 (0 if Q1, 0.333 if Q2, 0.666 if Q3, and 1 if Q4).

### Ethical Approval

This study does not involve identifiable human participants. The Universidad de Costa Rica (VI-428-2020) provided ethics approval. The National Institute of Statistics and Census approved the use of data after anonymization.

## RESULTS

Table 1 describes sample characteristics and cause of death. The two main causes of death were diseases of the circulatory system (30%) and neoplasms (25%). The mean age of death was 72 years old and was between 70 and 80 years old for the majority of the specific causes of death, under 70 years old for external causes of morbidity and mortality (53 years old), certain infectious and parasitic diseases (62 years old), and diseases of the musculoskeletal system and connective tissue (67 years old), and above 80 years old for mental and behavioral disorders (85 years old). Women were 44% of the deceased population and represented 40%-50% of the deaths from most specific causes of death. Moreover, the proportion of women was higher was higher proportion of diseases of the musculoskeletal system and connective tissue (69%), mental and behavioral disorders (57%), and endocrine, nutritional and metabolic diseases (53%). In turn, women were a particularly low proportion of external causes of morbidity and mortality (23%) and for Certain infectious and parasitic diseases (36%).

**Table 1:** Descriptive statistics of the sample (unweighted) (n = 2,739,733).

	n	%^a^	Mean age^d^	% women
Sex				
Men	1,366,435	50	50	-
Women	1,373,298	50	51	-
Death (all causes) (n)^c^	153,815	6	72	44
		**^b^**		
Certain infectious and parasitic diseases	2,866	2	62	36
Neoplasms	38,315	25	70	46
Endocrine, nutritional and metabolic diseases	8,217	5	73	53
Mental and behavioral disorders	2,420	2	85	57
Diseases of the nervous system	4,161	3	71	48
Diseases of the circulatory system	46,436	30	76	45
Diseases of the respiratory system	14,799	10	79	46
Diseases of the digestive system	12,154	8	70	43
Diseases of the genitourinary system	5,211	3	75	44
Diseases of the musculoskeletal system and connective tissue	1,493	1	67	69
Symptoms, signs and abnormal clinical and laboratory findings, not elsewhere classified	1,567	1	74	39
External causes of morbidity and mortality	14,785	10	53	23
Other	1,391	1	70	54

^a^ Percentage of the sample.

^b^ Percentage of the deaths.

^c^ Cause of death according to the 10^th^ revision of the International Classification of Diseases (ICD-10).

^d^ Mean age at the end of follow-up or at death.

Table 2 describes the causes of death by sex, urbanicity, and district wealth. The two main causes of death were diseases of the circulatory system and deoplasms, which together represent more than 50% of deaths in both men and women. The main difference in death distribution between men and women was the importance of the external causes of morbidity and mortality, which represented 13.3% of deaths in men (third cause of death) and only 5.0% of deaths in women (seventh cause of death). A similar distribution of causes of death can be observed across urbanicity and wealth. Death from external causes of morbidity and mortality was higher in rural districts (11.1%) than in urban districts (8.9%) and in poorer districts (13.0%) than in wealthier districts (7.4%). In turn, death from neoplasms and diseases of the circulatory system was higher in the wealthiest districts (26.6% and 31.6%, respectively) than in the poorest districts (23.7% and 27.6%, respectively).

**Table 2:** Distribution of specific causes of death by sex, area, and district wealth using the unweighted sample (unweighted) (n = 2,739,733).

	Sex		Area			District wealth			
	Men	Women	Rural	Mixed	Urban	Q1	Q2	Q3	Q4
Certain infectious and parasitic diseases	2.1%	1.5%	1.5%	1.7%	2.1%	1.8%	1.9%	1.9%	2.1%
Neoplasms	24.1%	26.0%	25.7%	24.0%	25.5%	26.6%	25.2%	24.1%	23.7%
Endocrine, nutritional and metabolic diseases	4.5%	6.5%	4.8%	5.7%	5.3%	5.0%	5.4%	5.7%	5.4%
Mental and behavioral disorders	1.2%	2.0%	1.5%	1.3%	1.7%	1.8%	1.6%	1.4%	1.2%
Diseases of the nervous system	2.5%	3.0%	2.6%	2.6%	2.9%	3.1%	3.0%	2.6%	2.2%
Diseases of the circulatory system	29.4%	31.2%	28.8%	29.3%	30.5%	31.6%	30.5%	29.5%	27.6%
Diseases of the respiratory system	9.2%	10.1%	10.7%	10.1%	9.0%	9.1%	9.2%	9.8%	10.1%
Diseases of the digestive system	8.0%	7.8%	7.3%	7.7%	8.1%	7.8%	8.4%	7.8%	7.7%
Diseases of the genitourinary system	3.4%	3.4%	3.2%	3.8%	3.2%	3.0%	3.1%	3.9%	3.8%
Diseases of the musculoskeletal system and connective tissue	0.5%	1.5%	0.8%	0.9%	1.0%	1.0%	1.0%	0.9%	1.0%
Symptoms, signs and abnormal clinical and laboratory findings, not elsewhere classified	1.1%	0.9%	1.3%	1.2%	0.9%	0.8%	1.0%	1.0%	1.4%
External causes of morbidity and mortality	13.3%	5.0%	11.1%	10.9%	8.9%	7.4%	8.9%	10.6%	13.0%
Other	0.7%	1.1%	0.9%	0.9%	0.9%	1.0%	0.9%	0.9%	0.9%

Q1 (wealthiest districts), Q4 (poorest districts).

Table 3 presents the results of the parametric survival models for the main chapters of the ICD-10 classification in men and women. Overall, the Relative Inequality Index increased after adjustment for urbanicity for most causes of death, showing that considering urbanicity allows better representing inequalities in Costa Rica.

**Table 3:** Parametric survival model adjusted for urbanicity and district wealth (n = 2,739,733).

Variables	Certain infectious and parasitic diseases	Neoplasms	Endocrine, nutritional and metabolic diseases	Mental and behavioral disorders	Diseases of the nervous system
	HR [95%CI]	HR [95%CI]	HR [95%CI]	HR [95%CI]	HR [95%CI]
Urbanicity					
^ ^Urban	1	1	1	1	1
Mixed	0.62 [0.53–0.72]	0.92 [0.89–0.95]	0.97 [0.87–1.07]	0.80 [0.69–0.94]	0.94 [0.86–1.03]
Rural	0.46 [0.38–0.56]	0.93 [0.89–0.98]	0.74 [0.64–0.85]	0.89 [0.72–1.10]	0.95 [0.83–1.08]
Wealth					
Q1 (richer)	1	1	1	1	1
Q2	1.12 [0.96–1.31]	1.02 [0.99–1.05]	1.19 [1.09–1.30]	1.10 [0.97–1.26]	1.06 [0.97–1.15]
Q3	1.37 [1.16–1.62]	1.04 [1.00–1.07]	1.34 [1.16–1.54]	1.02 [0.87–1.20]	0.95 [0.87–1.04]
Q4	1.82 [1.51–2.18]	1.02 [0.98–1.06]	1.35 [1.18–1.55]	0.93 [0.76–1.12]	0.82 [0.73–0.91]
Relative Index of Inequality					
RII_NA_	1.18 [1.02–1.37]	0.97 [0.94–1.00]	1.25 [1.13–1.39]	0.83 [0.73–0.95]	0.80 [0.74–0.87]
RII_A_	1.77 [1.47–2.14]	1.03 [0.99–1.07]	1.40 [1.21–1.61]	0.96 [0.80–1.16]	0.85 [0.77–0.93]
**Variables**	**Diseases of the circulatory system**	**Diseases of the respiratory system**	**Diseases of the digestive system**	**Diseases of the genitourinary system**	**Diseases of the musculoskeletal system and connective tissue**
	**HR [95%CI]**	**HR [95%CI]**	**HR [95%CI]**	**HR [95%CI]**	**HR [95%CI]**
Urbanicity					
Urban	1	1	1	1	1
Mixed	0.91 [0.88–0.95]	1.00 [0.92–1.08]	0.86 [0.81–0.92]	0.97 [0.83–1.13]	0.84 [0.73–0.96]
Rural	0.84 [0.79–0.89]	0.95 [0.86–1.04]	0.75 [0.68–0.81]	0.71 [0.59–0.85]	0.67 [0.53–0.84]
Wealth					
Q1 (richer)	1	1	1	1	1
Q2	1.09 [1.05–1.13]	1.15 [1.08–1.23]	1.17 [1.10–1.24]	1.15 [1.04–1.27]	1.15 [0.99–1.34]
Q3	1.11 [1.06–1.17]	1.25 [1.14–1.36]	1.18 [1.11–1.25]	1.53 [1.26–1.87]	1.08 [0.93–1.26]
Q4	1.08 [1.03–1.14]	1.30 [1.18–1.43]	1.23 [1.12–1.36]	1.60 [1.34–1.91]	1.35 [1.13–1.61]
Relative Index of Inequality					
RII_NA_	1.00 [0.96–1.04]	1.28 [1.20–1.37]	1.06 [0.99–1.13]	1.50 [1.33–1.68]	1.06 [0.92–1.22]
RII_A_	1.11 [1.05–1.17]	1.31 [0.19–1.45]	1.23 [1.13–1.34]	1.69 [1.39–2.05]	1.27 [1.07–1.50]
**Variables**	**Symptoms, signs and abnormal clinical and laboratory findings, not elsewhere classified**	**External causes of morbidity and mortality**	**Other**		
	**HR [95%CI]**	**HR [95%CI]**	**HR [95%CI]**		
Urbanicity					
Urban	1	1	1		
Mixed	1.04 [0.88–1.24]	0.93 [0.85–1.01]	0.93 [0.78–1.11]		
Rural	0.95 [0.75–1.21]	0.81 [0.72–0.90]	0.90 [0.70–1.15]		
Wealth					
Q1 (richer)	1	1	1		
Q2	1.29 [1.06–1.58]	1.15 [1.04–1.27]	0.98 [0.84–1.13]		
Q3	1.38 [1.14–1.68]	1.48 [1.32–1.68]	1.12 [0.89–1.41]		
Q4	1.83 [1.46–2.30]	1.74 [1.59–1.91]	1.09 [0.90–1.33]		
Relative Index of Inequality					
RII_NA_	1.77 [1.48–2.12]	1.61 [1.50–1.74]	1.06 [0.90–1.25]		
RII_A_	1.75 [1.42–2.17]	1.78 [1.61–1.97]	1.13 [0.90–1.42]		

Note: Model adjusted for sex. RII_NA:_ RII not adjusted for urbanicity; RII_A:_ RII adjusted for urbanicity.

The analysis of various causes of death showed a negative social gradient after adjustment for urbanicity where mortality decreased with district wealth for the following causes of death (Table 3): certain infectious and parasitic diseases (HR_Q4_ = 1.82 [1.51–2.18]); endocrine, nutritional and metabolic diseases (HR_Q4_ = 1.35 [1.18–1.55]); diseases of the respiratory system (HR_Q4_ = 1.30 [1.18–1.43]); diseases of the genitourinary system (HR_Q4_ = 1.60 [1.34–1.91]); and external causes of morbidity and mortality (HR_Q4_ = 1.74 [1.59–1.91]). Moreover, other causes had no clear social gradient but higher mortality in poorer districts than in wealthier districts, such as diseases of the circulatory system, diseases of the digestive system, and diseases of the musculoskeletal system and connective tissue. Neoplasms and mental and behavioral disorders, in turn, showed no clear relationship between district deprivation and mortality. Finally, diseases of the nervous system were the only cause of death with a positive social gradient (HR_Q4_ = 0.82 [0.73–0.91]).

Mortality was significantly higher in urban districts than in mixed and rural districts after adjustment for wealth for most of the main chapters of ICD–10, including certain infectious and parasitic diseases (HR_rural_ = 0.46 [0.38–0.56]), neoplasms (HR_rural_ = 0.93 [0.89–0.98]), endocrine, nutritional and metabolic diseases (HR_rural_ = 0.74 [0.64–0.85]), diseases of the circulatory system (HR_rural_ = 0.84 [0.79–0.89]), diseases of the digestive system (HR_rural_ = 0.75 [0.68–0.81]), diseases of the genitourinary system (HR_rural_ = 0.71 [0.59–0.85]), diseases of the musculoskeletal system and connective tissue (HR_rural_ = 0.67 [0.53–0.84]), and external causes of morbidity and mortality (HR_rural_ = 0.81 [0.72–0.90]). No difference by urbanicity was found for mand behavioral disorders, diseases of the nervous system, or diseases of the respiratory system. Finally, no ICD chapter had higher mortality in rural districts after adjustment for wealth.

Table 4 and Table 5 present the results of the parametric survival models for the main chapters of the ICD-10 classification in men and in women respectively. Women showed a clear positive social gradient for all causes of death except mental and behavioral disorders and diseases of the nervous system. Men, however, had no clear social gradient for the two mentioned causes and for endocrine, nutritional and metabolic diseases, diseases of the circulatory system, diseases of the digestive system, and diseases of the musculoskeletal system and connective tissue. For external causes of morbidity and mortality, men had a stronger positive social gradient than women.

**Table 4:** Parametric survival model adjusted for urbanicity and district wealth in men (n = 1,366,435).

Variables	Certain infectious and parasitic diseases	Neoplasms	Endocrine, nutritional and metabolic diseases	Mental and behavioral disorders	Diseases of the nervous system
	HR [95%CI]	HR [95%CI]	HR [95%CI]	HR [95%CI]	HR [95%CI]
Urbanicity					
Urban	1	1	1	1	1
Mixed	0.59 [0.49–0.71]	0.88 [0.84–0.92]	0.79 [0.69–0.90]	0.81 [0.66–0.99]	0.89 [0.80–1.00]
Rural	0.44 [0.34–0.56]	0.85 [0.81–0.90]	0.53 [0.44–0.64]	0.87 [0.68–1.12]	1.00 [0.85–1.19]
Wealth					
Q1 (richer)	1	1	1	1	1
Q2	1.09 [0.90–1.33]	1.01 [0.97–1.06]	1.04 [0.93–1.16]	1.02 [0.85–1.21]	1.07 [0.95–1.19]
Q3	1.32 [1.07–1.62]	1.01 [0.96–1.06]	1.16 [0.97–1.37]	0.92 [0.73–1.17]	0.98 [0.87–1.11]
Q4	1.68 [1.35–2.09]	0.97 [0.92–1.03]	1.10 [0.92–1.31]	0.92 [0.71–1.20]	0.84 [0.72–0.98]
**Variables**	**Diseases of the circulatory system**	**Diseases of the respiratory system**	**Diseases of the digestive system**	**Diseases of the genitourinary system**	**Diseases of the musculoskeletal system and connective tissue**
	**HR [95%CI]**	**HR [95%CI]**	**HR [95%CI]**	**HR [95%CI]**	**HR [95%CI]**
Urbanicity					
Urban	1	1	1	1	1
Mixed	0.88 [0.85–0.92]	0.91 [0.84–0.99]	0.81 [0.74–0.88]	0.96 [0.78–1.18]	0.79 [0.64–0.98]
Rural	0.81 [0.75–0.86]	0.81 [0.73–0.91]	0.65 [0.57–0.74]	0.67 [0.52–0.85]	0.62 [0.42–0.90]
Wealth					
Q1 (richer)	1	1	1	1	1
Q2	1.06 [1.02–1.10]	1.11 [1.03–1.20]	1.12 [1.14–1.21]	1.11 [0.98–1.25]	0.91 [0.70–1.18]
Q3	1.06 [1.01–1.12]	1.21 [1.10–1.33]	1.08 [0.99–1.17]	1.67 [1.29–2.17]	0.97 [0.76–1.23]
Q4	1.02 [0.96–1.07]	1.25 [1.12–1.40]	1.13 [0.99–1.27]	1.62 [1.28–2.05]	1.02 [0.76–1.36]
**Variables**	**Symptoms, signs and abnormal clinical and laboratory findings, not elsewhere classified**	**External causes of morbidity and mortality**	**Other**		
	**HR [95%CI]**	**HR [95%CI]**	**HR [95%CI]**		
Urbanicity					
Urban	1	1	1		
Mixed	1.05 [0.88–1.26]	0.90 [0.82–1.00]	0.85 [0.68–1.25]		
Rural	0.90 [0.70–1.15]	0.77 [0.68–0.87]	0.96 [0.68–1.37]		
Wealth					
Q1 (richer)	1	1	1		
Q2	1.40 [1.11–1.77]	1.20 [1.07–1.35]	1.02 [0.82–1.28]		
Q3	1.55 [1.25–1.91]	1.62 [1.42–1.84]	1.26 [0.94–1.67]		
Q4	2.02 [1.60–2.55]	1.95 [1.75–2.16]	0.92 [0.68–1.25]		

**Table 5:** Parametric survival model adjusted for urbanicity and district wealth in women (n = 1,373,298).

Variables	Certain infectious and parasitic diseases	Neoplasms	Endocrine, nutritional and metabolic diseases	Mental and behavioral disorders	Diseases of the nervous system
	HR [95%CI]	HR [95%CI]	HR [95%CI]	HR [95%CI]	HR [95%CI]
Urbanicity					
Urban	1	1	1	1	1
Mixed	0.69 [0.59–0.82]	0.96 [0.92–1.00]	1.16 [1.05–1.28]	0.81 [0.68–0.93]	1.01 [0.89–1.14]
Rural	0.54 [0.42–0.69]	1.02 [0.96–1.10]	0.98 [0.83–1.15]	0.93 [0.70–1.25]	0.88 [0.72–1.08]
Wealth					
Q1 (richer)	1	1	1	1	1
Q2	1.18 [0.96–1.45]	1.01 [0.97–1.05]	1.33 [1.19–1.49]	1.17 [1.00–1.37]	1.05 [0.93–1.19]
Q3	1.48 [1.24–1.76]	1.05 [1.01–1.10]	1.52 [1.32–1.74]	1.10 [0.91–1.33]	0.93 [0.81–1.06]
Q4	2.19 [1.75–2.72]	1.07 [1.00–1.14]	1.66 [1.44–1.91]	0.91 [0.72–1.16]	0.81 [0.69–0.96]
**Variables**	**Diseases of the circulatory system**	**Diseases of the respiratory system**	**Diseases of the digestive system**	**Diseases of the genitourinary system**	**Diseases of the musculoskeletal system and connective tissue**
	**HR [95%CI]**	**HR [95%CI]**	**HR [95%CI]**	**HR [95%CI]**	**HR [95%CI]**
Urbanicity					
Urban	1	1	1	1	1
Mixed	0.97 [0.93–1.02]	1.12 [1.01–1.24]	0.96 [0.88–1.05]	0.98 [0.85–1.13]	0.86 [0.73–1.02]
Rural	0.91 [0.85–0.97]	1.16 [1.03–1.30]	0.92 [0.81–1.04]	0.79 [0.65–0.94]	0.69 [0.53–0.91]
Wealth					
Q1 (richer)	1	1	1	1	1
Q2	1.13 [1.08–1.18]	1.20 [1.10–1.31]	1.23 [1.13–1.34]	1.20 [1.05–1.38]	1.27 [1.08–1.50]
Q3	1.17 [1.11–1.24]	1.28 [1.13–1.44]	1.32 [1.20–1.45]	1.37 [1.16–1.63]	1.13 [0.93–1.37]
Q4	1.20 [1.12–1.29]	1.37 [1.21–1.56]	1.42 [1.26–1.61]	1.59 [1.33–1.91]	1.54 [1.23–1.92]
**Variables**	**Symptoms, signs and abnormal clinical and laboratory findings, not elsewhere classified**	**External causes of morbidity and mortality**	**Other**		
	**HR [95%CI]**	**HR [95%CI]**	**HR [95%CI]**		
Urbanicity					
Urban	1	1	1		
Mixed	1.07 [0.82–1.39]	1.07 [0.98–1.17]	1.00 [0.81–1.24]		
Rural	1.13 [0.77–1.65]	1.03 [0.89–1.18]	0.82 [0.59–1.15]		
Wealth					
Q1 (richer)	1	1	1		
Q2	1.20 [0.90–1.59]	1.08 [0.98–1.18]	0.93 [0.77–1.12]		
Q3	1.21 [0.91–1.60]	1.17 [1.05–1.30]	1.00 [0.78–1.28]		
Q4	1.67 [1.17–2.40]	1.31 [1.16–1.47]	1.27 [0.98–1.63]		

Differences according to urbanicity were more common in men than in women after adjustment for wealth. In men, mortality was higher in urban districts than in rural districts for all causes except for mental and behavioral disorders and diseases of the nervous system. In women, only four causes of deaths showed a higher mortality in urban districts than in rural districts: certain infectious and parasitic diseases, diseases of the circulatory system, diseases of the genitourinary system, and diseases of the musculoskeletal system and connective tissue. Diseases of the respiratory system showed conflicting results, where, compared to urban districts, mortality was lower in rural districts for men and higher for women.

## DISCUSSION

After adjustment for urbanicity, the poorer districts of Costa Rica presented a higher mortality than the wealthier districts for most causes of death, except for neoplasms, mental and behavioral disorders, and diseases of the nervous system. Moreover, after adjustment for wealth, urban districts had significantly higher mortality than mixed and rural districts for most ICD-10 classifications, except for mental and behavioral disorders, diseases of the nervous system, and diseases of the respiratory system. Differences according to wealth were more frequent in women than in men, whereas differences according to urbanicity were more frequent in men than in women.

These results allowed better understanding the findings of a previous study which used the same methodology^33^. In this previous study, we observed a clear negative social gradient in mortality after 20 years of age for men and women in both rural and urban districts, where mortality was lower in the wealthiest urban districts than in the poorest urban districts and lower in the wealthiest rural districts than in the poorest rural districts^33^. In men, however, this negative social gradient weakened after 60 years of age^33^.

Furthermore, mortality was lower in rural districts than in urban districts for men but not for women^33^. In our study, district wealth was more often associated with mortality from specific causes in women than in men, whereas urbanicity was more associated with mortality in men than in women. In women, most causes of death had a negative social gradient, but urbanicity was not associated with all the causes. In men, rural districts presented lower mortality than urban districts, but various causes showed no social gradient – except for external causes of morbidity and mortality, which showed a significant negative social gradient in men.

This result corroborates two previous studies in Costa Rica, which showed inequalities in this cause of death^13^ and the importance of vehicle accidents and homicides to explain life expectancy differences according to province for men but not for women^32^. Moreover, it explains why the social gradient was more important for men at 20 years old than at 60 years old and why the mean age of death from external causes of morbidity and mortality is low.

This study showed that, in Costa Rica, most causes of death present a social gradient, corroborating the international literature. However, the two main causes of death, neoplasms and diseases of the circulatory system, showed a social gradient in women, but not in men. This result is unexpected since these two causes are relevant to explain inequalities in life expectancy in the international literature^8,11^. Moreover, Rosero-Bixby and Dow showed inequalities also in cerebrovascular mortality^13^ in Costa Rica.

The main causes for inequality were those closely related to socio-economic characteristics and risky health behaviors (e.g. smoking and alcohol consumption)^14^. Describing the distribution of risky health behaviors according to urbanicity and socioeconomic indicators is thus essential to contextualize the results of our study. In Costa Rica, smoking prevalence is low, especially for women (4%)^37^, and alcohol consumption is lower than in the United States or Europe^38^. These two factors could thus affect the socioeconomic characteristics of the population less than in other countries. In men, smoking prevalence is lower in rural areas than in urban areas, but it is weakly-linked to socioeconomic status^37^. Studies suggest that alcohol consumption has a positive social gradient, where the privileged drink more than the underprivileged^39,40^. In adults, obesity prevalence is lower in rural areas than in urban areas for men^41^, but not for women. In children, overweight and obesity is lower in rural and poorer districts than in urban and wealthier districts^42^.

Our study has limitations. Our results could not be fully explained because of the lack of data on behavior distribution according to social class in Costa Rica. Individual information on behaviors, occupation, housing conditions, or access to the health system would have allowed us to better understand how each characteristic contributes to mortality inequality. Children and foreigners were excluded from the study. Since child mortality is low in Costa Rica, excluding children should not create bias. Nevertheless, as foreigners usually have more difficulty to access the health system^22^, they might present a different mortality pattern. Another limitation is that using the electoral district is not as ideal as using individual measures of socioeconomic position. Moreover, some people might not be registered in the NRE with their latest address. We had to weight the sample to consider the 5% loss of the death certificates, supposing that the variables used to calibrate (sex, age, district characteristics, cause of death) were sufficient to avoid bias. Finally, only the main cause of death was included on the death certificates.

Our study had several strengths. The National Electoral Rolls and the National Death Index are nearly complete^27,32^, which allowed us to follow all the Costa Rican citizens for nine years using the electoral district, an official indicator of residence. District characteristics were based on census data, which ensures their precision. Using a survival model allowed us to consider deaths from other causes as competitive risks for each specific cause of death. Finally, the sample size of over 153,000 deaths allowed us to analyze the relation between the main chapter of ICD-10 and socioeconomic characteristics of the districts in an ecological study. This represents an important step for studying social inequalities in mortality in low- and middle-income countries.

For most ICD-10 chapters, the poorest districts had higher mortality than the wealthiest districts after adjustment for urbanicity whereas urban districts had higher mortality than mixed and rural districts after adjustment for wealth. Our results also showed differences between men and women, where wealth characteristics of the district were more significant in women and urbanicity was more significant in men. These results were consistent but partly different from the literature in high-income countries. In particular, women presented a negative social gradient for the two main causes of deaths, neoplasms and diseases of the circulatory system, corroborating the international literature. However, men did not present these gradients, reinforcing the importance of reporting evidence from different contexts – especially in middle-income countries. Detailing the causes of death helps identify which causes are related to the greatest socioeconomic gaps and implement specific public health measures adapted to each country.
